# Supplementary material for: COVID-19 vaccine hesitancy: Vaccination intention and attitudes of community health volunteers in Kenya
Source: PLOS Glob Public Health. 2022 Mar 16;2(3):e0000233. doi: 10.1371/journal.pgph.0000233 (PMC10021929; doi:10.1371/journal.pgph.0000233)
Supplement: S1 Questionnaire — (DOCX) [file pgph.0000233.s001.docx]

# **CROSS-SECTIONAL SURVEY QUESTIONS FOR COMMUNITY HEALTH VOLUNTEERS**

1. **Demographic Data**
2. **County**
3. **Sex**  A. Male B. Female C. Other
4. **Age** A. 18 – 24 years B. 25 – 35 years C. Above 35 years
5. **Religion** A. Catholic B. Protestant C. Islam D. others
6. **Highest education level completed**
   1. None B. Primary C. Secondary D. College/university
7. **Years of services as a CHV** A. Less than 3 B. 3 – 5 years C. Above 5 years
8. **Number of households you are responsible for**
   1. 20 or less b. 21 – 50 c. More than 50
9. **Have you had MoH approved training on COVID-19?**

A. Yes B. No C. Not sure

1. **Have you been involved in educating the community on COVID-19?**
   1. Yes B. No
2. **Your main source of income**
   1. CHV work B. Other formal employment C. Other non-formal employment
3. **Psychographic Data**
4. **Attitude: Contextual Influences – Historical, socio-cultural, health system, environmental, economic, political**
5. What is the most common source of information on COVID-19 vaccination that you and your community come across?

A. Social media B. Radio/TV C. IEC materials from MoH D. Community meetings E. Other (Explain)……………………………….

1. When you come across negative information on COVID-19 vaccine who do you turn to for verification?

A. Friends/relatives B. Internet C. Fellow health workers D. Other (Explain) ………………………………………………………………………

1. Do you remember any vaccine-associated health problems in the past that may prevent you from getting the COVID-19 vaccine?
   1. Yes (Explain)…………………………………………………………………………………………………………
   2. No
2. Do you know people in your community who oppose COVID-19 vaccine on religious grounds? A. Yes B. No

If yes, explain your answer above ………………......................

1. Do you know people in your community who oppose COVID-19 vaccine on cultural grounds? A. Yes B. No

If yes, explain the cultural reasons why people in your community oppose the COVID-19 vaccine ……………………………………………………………………………………………

1. In your view is the MoH making the right decisions on COVID-19 vaccination?
   1. Yes B. No

Explain your answer above ……………………………………………………………………………

1. Do you think vaccine manufacturers have good intentions for you and people in your community?
   1. Yes B. No

Explain your answer above ……………………………………………………………………….. ..………………………………..……………………………………………………………………………………..

1. **Attitude: Individual and Group Influences**
2. I) Are you aware of any bad reactions in people who have had COVID-19 vaccination?
   1. Yes B. No

II) If yes, explain your answer ………………………………………………………………………………….

1. I) Do you believe that there are other better ways to prevent COVID-19 than giving people the vaccine?
   1. Yes B. No

II) If yes, explain your answer………………………………………………….

1. I) Do you feel that information on COVID-19 vaccines is being openly shared?
   1. Yes B. No
      1. If no, why is information on COVID-19 vaccine not being openly shared?

1. Do you trust what the MoH says about COVID-19 vaccination?
   1. Yes B. No
2. In your view is the COVID-19 vaccine safe enough for people to be injected?
   1. Yes B. No
3. Do you support mass vaccination (vaccinating everyone) with COVID-19 vaccine?
   1. Yes B. No
4. **Attitude Questions: Vaccine safety/Vaccination Specific Issues**
5. Do you feel our country can manage risks associated with COVID-19 vaccine side effects?

A. Yes B. No

1. Can the health system be trusted to deliver COVID-19 vaccine to your communities
2. Yes B. No
3. How confident are you in the safety of COVID-19 vaccine?

A. Very confident B. Confident C. Somehow confident D. Not confident E. Not confident at all F. Don’t know

1. How concerned are you that the COVID-19 vaccine might not be safe for the public?

A. Not at all B. Not too much C. Somewhat D. Very much E. Don’t know

1. What concerns if any do you have about COVID-19 vaccine?
   1. Vaccines are not tested enough for safety
   2. The ingredients used to make the COVID-19 vaccine are unsafe
   3. The COVID-19 vaccine might weaken the immunity of the people
   4. Vaccine is only given to people who are seriously infected with the virus
   5. People cannot be vaccinated for a disease they do not have
   6. The vaccine might cause chronic diseases
   7. The COVID-19 vaccine might be very expensive for many people to afford
   8. The government might not be able to ensure enough supply of the vaccine and therefore result in a more negative effect from the disease compared to when the vaccine had not been introduced
   9. I don’t have any concerns about the COVID-19 vaccine
2. In general, how safe do you think COVID-19 vaccine is for the general population?
   - 1. Not at all B. Not too much C. Somewhat D. Very much E. Don’t know

**Vaccination Intention Questions**

1. I am planning to get the COVID-19 vaccination once it’s available in the country

Strongly Disagree (1): Disagree (2) : Neutral (3): Agree (4): Strongly Agree (5)

1. I intend to get the COVID-19 vaccination once it’s available in the country

Strongly Disagree (1): Disagree (2) : Neutral (3): Agree (4): Strongly Agree (5)

1. I will try to get the COVID-19 vaccination once it’s available in the country

Strongly Agree (1): Agree (2) : Neutral (3): Disagree (4): Strongly Disagree (5)
