# Supplementary material for: COVID-19 vaccine hesitancy: Vaccination intention and attitudes of community health volunteers in Kenya
Source: PLOS Glob Public Health. 2022 Mar 16;2(3):e0000233. doi: 10.1371/journal.pgph.0000233 (PMC10021929; doi:10.1371/journal.pgph.0000233)
Supplement: S1 File — Informed consent for cross-sectional survey. (DOCX) [file pgph.0000233.s002.docx]

# **CONSENT FORMS**

# **Informed consent for Cross-sectional survey**

**Introduction and purpose of the study**

Amref Health Africa is conducting a study in four counties namely Nairobi, Mombasa, Trans-Nzoia, and Kajiado, Kenya. The purpose of this study is to assess the knowledge, attitude and acceptance of COVID-19 vaccine among community health volunteers in Mombasa, Nairobi, Kajiado, and Trans-Nzoia Counties in Kenya. The information gathered will be analyzed and evaluated to make recommendations for rolling out a COVID-19 vaccination programme in Kenya.

**Interview Participant Selection**

Community health volunteers who work in the Sampled Sub-counties of Nairobi, Mombasa, Kajiado and Trans-Nzoia Counties are eligible to participate in the quantitative survey. You are being invited to take part in this research project because we feel that your knowledge, attitude and perception of COVID-19 vaccine as a community health volunteer will assist us to know the best entry point for planning a vaccination programme.

**Voluntary Participation**

Your participation in this research is completely voluntary. You will make the choice about whether you will participate or not. If you choose not to take part, there will be no negative consequences.

**Study Procedure**

The survey will use an interviewer administered questionnaire. The interviewer will record your responses onto the questionnaire, and will also make an audio recording of the discussion. The interview will take approximately 30-45 minutes to complete. During the interview, the interviewer will ask questions on your work or volunteer experience and you are urged to share any information that might improve the service delivery to the community. Should you have questions at any stage of the interview, feel free to ask at any time.

**Study Duration:** This study is going to take 6 months.

**Potential Risks**

There are no known physical risks associated with this study. However, there is a risk that you may share some personal or confidential information by chance, or that you may feel uncomfortable talking about some of the topics in this study. You do not have to answer any question or take part in the survey if you feel the question(s) are too personal or if talking about them makes you uncomfortable, and you can stop the interview at any time.

**Potential Benefits**

There will be no direct benefit to you, but your participation is likely to help us to get the knowledge level, attitude, and acceptance level of the COVID-19 vaccine at community level, and therefore help to inform planning of a national vaccination programme in Kenya.

**Confidentiality**

The information that we collect from this research project will be kept private. Questionnaires and any other materials for this survey will be identified by coded numbers and your name will never appear. The only record that would identify you by name is this Consent Form, and this will be kept under lock and key in the investigator’s office and not allowed to leave the investigator’s office. Only the study personnel will have access to the information records. All the data will be kept secure under lock and key and in a password protected computer and will be accessible to the researchers only, all of whom have signed a confidentiality agreement.

**Dissemination of Results**

The findings from this research will be shared with the County Health Management team, and you will be able to access this information from the County and sub-County Health Offices. The study findings will be shared with other implementing partners and will also be presented and published in other forums so that other interested people may learn from the research.

**Withdrawal from the Study**

You do not have to take part in this research if you do not wish to. If you choose not to participate, there will be no negative consequences. If you wish to stop participating in the study after you begin, you can stop at any time by telling someone on our project team. If you choose to stop taking part, there will be no negative consequences.

**Contact Information for Concerns about the Rights of Research Subjects**

If you have any questions, you can ask anyone from our team now or later. If you have questions later, you may contact …………………………… If you have questions about your rights as a research participant, you may contact:

Principal Investigator- Professor Joachim Osur

Amref Health Africa in Kenya,

Mobile - 0718766864

Co-investigator- Evelyne Muinga

Amref Health Africa in Kenya,

Mobile - 0720063143

Co-investigator- Edward Ireri

Amref International University, Kenya,

Mobile – 0725812728

Co-investigator- Dr. Jan Carter,

Amref Health Africa in Kenya,

Mobile- 0788481541

Co-investigator- Kuria Shiphrah

Amref Health Africa in Kenya,

Mobile – 0722300279

Co-investigator- Salim A. Hussein

Ministry of Health Kenya,

Mobile - 0728136555

**Do you have any questions at this time?**

**Part II: Certificate of Consent**

I have read this form and I freely consent to participate in this study.

Participant’s name Participant’s Signature Date

A copy of this ICF has been provided to the participant.

Interviewer’s name Interviewer’s Signature Date

**Informed consent for the Key Informant Interviews**

Thank you for accepting to be a part of this key informant interview. My name is ___________ and I will be leading the discussion session. My colleague _________ will assist with taking the discussion notes. We are here today on behalf of the Amref Health Africa and Ministry of Health to collect information about COVID-19 vaccine. Specifically, we would like to have a discussion with you, to get your views and perception on the COVID-19 vaccine, so as to know the entry point to plan for a COVID-19 vaccination programme in Kenya. There are no right or wrong answers. Your participation is voluntary, and you are free to withdraw from the interview any time you don’t feel like continuing with the interview. Please I request for your cooperation.

**Risks/ benefits and discomforts**

The study involves questions which will require you to give your personal views which may make you feel uncomfortable. But your answers are important to inform the programme and help to plan for an effective vaccination programme in Kenya. From the findings of the study, you and your community members may benefit from long term programmes that are likely to be rolled out in this region. Please note that there will be no payment for participating in the study.

**Confidentiality**

Please note that the information which you provide will not be disclosed to anyone who is not part of the research team. Codes, not names, will be used in the research transcripts, while names shall be omitted in the transcription of audio recording. The recorders will be secured using locks to prevent any access by any non-research team. The information obtained from you shall be kept private and will solely be used for study purposes. Any record relating to your identity, such as name will not be disclosed. The information collected will be erased from the computers after analysis and publications of the results. Researchers will only be able to access the information about the research after they have signed a confidentiality agreement.

**Time of participation**

This discussion will take approximately one hour. Do you have questions at this point about this discussion?

If there are no questions, we can begin…
