# Supplementary material for: COVID-19 vaccine hesitancy: Vaccination intention and attitudes of community health volunteers in Kenya
Source: PLOS Glob Public Health. 2022 Mar 16;2(3):e0000233. doi: 10.1371/journal.pgph.0000233 (PMC10021929; doi:10.1371/journal.pgph.0000233)
Supplement: Attachment — Submitted filename: Response to Reviewers Comments.docx [file pgph.0000233.s003.docx]

| SECTION | REVIEWER #1 COMMENTS | AUTHORS RESPONSE |
| --- | --- | --- |
| Introduction | Efficacy and technologies of all vaccines are not required to be discussed in detail. | Paragraph on efficacy deleted and the one on technologies paraphrased. See page 3 |
|  | Instead of listing different names used for community health volunteer, their roles and responsibilities can be mentioned and include why their hesitancy needs to be studied? | Done. See page 4 |
|  | Kindly delete the table with literature review; rather discuss the key findings from previous studies in introduction. | Done. See pages 5 – 7. Incorporated on the introduction |
| Materials and methods | There are 47 counties in Kenya. How these four were selected? Sampling method? | Done. See pages 11-12. Study design |
|  | Please mention the parameters considered for sample size calculation | Done. See pages 12-13. Sample size determination |
|  | Data collection details are not provided. Who? Where? and How? | Done. See page 14. Data collection process |
|  | How was it possible to complete data collection within few days? | Done. See page 14. Data collection process. With a sample size of 408 and 11 RA’s, then on average, each public health official was supposed to make 37 calls within one week. |
|  | Kindly describe about the data collection tools. | Done. See page 14. Data collection tool. |
|  | First bar chart needs to be improved. It should be numbered. Axis lables to be added. | Done. See page 16. Axis labels have been added. |
| Inferential statistics | Distribution can be written after mentioning the prevalence with numbers. | Done. See page 20. |
|  | Table 2 can be restructured to make it less crowded. Demographic variables as rows and counties as columns. | Done. See page 33, Table 2. |
|  | What is R2 value for the goodness of fit? | Done. See Table 1 page 16 |
|  | What factors were considered for four models? | Done. See pages 15-17 |
|  | Independent variable being a categorical variable, is it required to present beta-coefficients? | Done. See Table 2, page 22. |
| Discussion | “Insignificant socio-demographic factors in vaccination intention were gender (contrary to [15] study in the USA; [19] study in Canada; [28] study in Saudi Arabia; [29] study in Saudi Arabia; [32]study in Australia; [39] and study in the UK); age (contrary to [31] in Bangladesh; [32] study in Australia; [39] study in the UK), religion (contrary to [32] study in Australia), years of service as a CHV, the number of households each CHV was attached to, and source of income (contrary to [15] study in the USA; [22] study in Colombia; [31] study in Bangladesh; [32] study in Australia;and [39] study in the UK)”. – need to be summarized and rewritten | Done. See page 29 |
|  | Strengths and limitations of the study are not mentioned. Kindly mention it. | Done. See page 32 |
| Recommendations | “This study recommends training CHVs on COVID-19 and its vaccines to reduce hesitancy, and funds for training must accompany the vaccines” the prevalence of vaccine acceptance is 81% which is very high. Therefore is the above recommendation appropriate? | Yes, the statement holds water. The study was conducted in March 2021. At the time of revising this manuscript, the uptake of Covid-19 vaccine was very high during the first dose (97%). Vaccine hesitancy for the second dose is very high, with the uptake for the second dose standing at 3%. Thus, training of CHV and building on trust are now very important to increase the uptake of the second dose. |
| Conclusion | “A strong association exists between CHVs’ vaccination intention and their readiness to engage with communities.” I couldn’t see any data suggestive of your conclusion?! Please make conclusions based on your findings. | Done. See page 35 under the section on the conclusion |
| SECTION | REVIEWER #2 COMMENTS | AUTHORS RESPONSE |
| 1. Objectives | The objectives of the study done were to find out vaccination intention, attitude of the Community Health Volunteers (CHVs) and their potential effect on vaccine roll out. The authors reported vaccination intention and factors associated with. | The question is not clear. However, the study intended to investigate VACCINATION INTENTION because there were no COVID-19 vaccines available in the country at the time of the study. Thus, the study was intended to come up with intervention measures that would help in increasing the uptake of the vaccine once made available. Thus, it was important to investigate the influence of attitude on vaccination intention in readiness for the vaccine rollout.  However, the study objective has been added on page 7, the last paragraph under the introduction section. |
| Results | From results section it appears that attitude was measured by factors associated with vaccination intention. | No. Vaccination intention was the outcome variable, while the three levels of attitude (contextual influences, individual and group influences and vaccination specific issues) formed the predictor variables. |
| Material & methods | Materials and methods section does not mention how attitude or potential effect on vaccine roll out was measured. | Done. See page 14 and 15 on description of data variables |
| 2. Data collection tool | Nothing is written about data collection tool which was used to gather the requisite information. It appears some interview was done with study participants, but details of the data collection tool need to be mentioned including the mode of collection. | Done. See page 14. Data collection process. |
| 3. Krejcie Morgan Formula | The authors have used Krejcie Morgan Formula for sample size calculation. This formulae, is used for sample size calculation for a finite population. Some assumptions are needed for which are, population size, confidence level, degrees of freedom, margin of errors. None of these were mentioned in the article. | Done. See pages 12-13. Sample size determination |
| 4. Figures | There are seven figures in the article. The figures are not explanatory and may be presented in table form. | Done. Additional narratives have been added to describe the alluvial diagrams and thus, the figures are retained. |
| SECTION | REVIEWER #3 COMMENTS | AUTHORS RESPONSE |
| 1. Introduction | a. The authors have talked about the spread of COVID in the first paragraph which sets platform for the study. The second paragraph and third paragraph which talks about the type of vaccine and their final details is unwarranted to this context. | Paragraph on efficacy deleted and the one on technologies paraphrased. See page 3 |
|  | b. What is not clear to me is the prevailing issues which led the authors to undertake this study-needs a strong justification backed with facts and figures | Done. See page 4 & 5 on the role and responsibility of community health volunteer in Kenya. One of the main mandates of CHV is to support vaccination services and there is a policy to that effect. |
|  | c. What was the magnitude of COVID morbidity and mortality in Kenya? Other African countries? | The focus of the study was on the COVID-19 vaccination. Although the number of positive covid-19 cases and deaths was high, one of the measures that the government of took was to vaccinate it people so that the economy could be opened up. As at the time of reviewing this manuscript, the morbidity and mortality figures have increased compared to the figures that were available during the start of the study. |
|  | d. Was there equitable distribution of vaccines all over the continent? Countries? | The authors think this question is not related to the topic. The study was to help the Kenya prepare for the vaccination activity. At the time of the study, no vaccine was available in the country |
|  | e. “Based on systematic literature reviews and interviews with immunization managers, attitudes as determinants of vaccine hesitancy were grouped into contextual influences, individual and group influences, and vaccine or vaccination specific issues.” – Needs further explanation. Who and why did they adapt this approach? | The strategic advisory group of experts (SAGE), is the principal advisory group to WHO for vaccines and immunization which was established in 1999. |
|  | f. There is lot of description on community health volunteers but lacks strong justification on why they were chosen. | Done. See page 4 |
|  | g. Summary of the published studies in table? What was the purpose of getting it in introduction? The same could have been explained in sentences. Probably, the focus should have been African countries. | Done. See pages 5 – 7. Incorporated on the introduction. There was limited literature on covid-19 vaccination intention and hesitancy in Africa. The team agreed to reference only peer reviewed papers. |
|  | h. There is no research question. There are no objectives. | Done. See last paragraph under the introduction on page 7 |
| 2. Methods | a. Study setting: Need to describe about the country Kenya and its counties. Literacy rate? Population? Access to health care facilities? Availability of vaccines? | Done. See page 14. Description of the study setting, the 11 sub-counties, and the role of public health officials in the study. |
|  | b. Hypothesis? | The study was an exploratory study and thus, the investigators wanted to explore the broader question and not to test/confirm the hypotheses. |
|  | c. What was the sampling frame? How was sampling done? Describe sample size calculation? Target population? | Done. See pages 11-12. Study design and pages 12-13 on sample size determination |
|  | d. What were the tools for data collection? Who, when and how were they collected? How was data validation done? Pilot study? Training? Issues? | Done. See page 14. Data collection tool. The ‘who’ see page 14, the ‘when’ see page 12 on the target population, and the ‘how’ & training see page 14 and 15. No pilot study was conducted. The study team had the mobile numbers for all the study participants. Data valiadation was randomly conducted on a daily basis and continuous basis using follow-up calls. |
|  | e. What data variables are collected? Operational definition for each of these variables needs to be specified. | Done. See page 14 and 15 on description of data variables |
|  | f. Data analysis: Needs to be specific and assumptions made needs justification? | Done. See pages 15-17 |
|  | g. Why are results discussed in data analysis?? | Done. See last paragraph page 25 |
| 3. Results | a. The results are complex and poorly described. Though, alluvial diagrams are attractive they fail to convey the messages. The country should be described in totality rather than individual counties. | Done. The alluvial diagrams have been described. See page 23, 24, 26 and 27 |
|  | b. Table 3 and 4 are not appropriately structured. It seems to be a output window of analysis. | Done: See Table 2. The previous tables were drawn using the format ‘html’ in stargazer package in R. It’s a package that is purely designed to generate tables in latex format, html format or pdf format. |
|  | c. The result section needs to be re-written | Done. The statistical analysis section has been beefed up including the model fit and its evaluation and the various statistical packages. (see pages 16-19). The results section has been expanded and re-written to include the actual p values, a new figure 1, a new table 2, the description of the alluvial diagrams (see pages 20-29) |
| 4. | a. The discussion lacks focus. Though, the authors have compared the findings with other studies; they have not appropriately reasoned out the current findings | We did not understand this question |
|  | b. Programmatic implications on the study findings | Done. See the section named recommendations and practical implications |
|  | c. Strengths and limitations of the study | Done. See page 32 |
|  | d. It is advisable to restructure and rewrite the discussion section. | We would like to maintain the current structure on the sub-section so that it becomes easier for any layman to follow the content of the manuscript |
