# Supplementary material for: COVID-19 vaccine hesitancy: Vaccination intention and attitudes of community health volunteers in Kenya
Source: PLOS Glob Public Health. 2022 Mar 16;2(3):e0000233. doi: 10.1371/journal.pgph.0000233 (PMC10021929; doi:10.1371/journal.pgph.0000233)
Supplement: Attachment — Submitted filename: Reviewer Comments Response.docx [file pgph.0000233.s004.docx]

Reviewer Response

We've checked your submission and before we can proceed, we need you to address the following issues

1. Tables should not be uploaded as individual files. Please remove these files and include the tables in your manuscript file.

*Response: Done. Tables have been inserted in their respective place in the manuscripts and removed from the system.*

2. We noticed that your Figures 3 & 4 and 5 & 6 are in one tif file. Please upload them separately in individual tif image.

*Response: Done. Figures 3 & 4 and 5 & 6 have been separated. They are now stand-alone figures.*

3. Please note that your Data Availability Statement is currently missing the DOI/accession number of each dataset OR a direct link to access each database.

Response: The data associated with the manuscript has been deposited on the Mendeley database. *See the link on page 11*

4. Please ensure that the funders and grant numbers match between the Financial Disclosure field and the Funding Information tab in your submission form. Note that the funders must be provided in the same order in both places as well. Currently, not all receiving authors are included Funding Information section.

*Response: This grant does not have a funding number. It was a donation from a corporation, which was not won through a competitive process, so there’s not even a call for proposals (or something like that) and thus no grant number.*
